# Robust Human-to-Robot Handover System Under Adverse Lighting

**DOI:** 10.3390/biomimetics11040231

**Published:** 2026-04-01

**Authors:** Yifei Wang, Baoguo Xu, Huijun Li, Aiguo Song

**Affiliations:** State Key Laboratory of Digital Medical Engineering, Jiangsu Key Laboratory of Robot Perception and Control, School of Instrument Science and Engineering, Southeast University, Nanjing 210096, China; 230219243@seu.edu.cn (Y.W.);

**Keywords:** human-to-robot handovers, 3D semantic segmentation, 6-DoF grasping

## Abstract

Human-to-robot (H2R) handovers are critical in human–robot interaction but are challenged by complex environments that impact robot perception. Traditional RGB-based perception methods exhibit severe performance degradation under harsh lighting (e.g., glare and darkness). Furthermore, H2R handovers occur in unstructured environments populated with fine-grained visual details, such as multi-angle hand configurations and novel object geometries, where conventional semantic segmentation and grasp generation approaches struggle to generalize. To overcome lighting disturbances, we present an H2R handover system with a dual-path perception pipeline. The system fuses perception data from a stereo RGB-D camera (eye-in-hand) and a time-of-flight (ToF) camera (fixed scene) under normal lighting, and switches to the ToF camera for reliable perception under glare and darkness. In parallel, to address the complex spatial and geometric features, we augment the Point Transformer v3 (PTv3) architecture by integrating a T-Net module and a self-attention mechanism to fuse the relative positional angle features between human and robot, enabling efficient real-time 3D semantic segmentation of both the object and the human hand. For grasp generation, we extend GraspNet with a grasp selection module optimized for H2R scenarios. We validate our approach through extensive experiments: (1) a semantic segmentation dataset with 7500 annotated point clouds covering 15 objects and 5 relative angles and tested on 750 point clouds from 15 unseen objects, where our method achieves 84.4% mIoU, outperforming Swin3D-L by 3.26 percentage points with 3.2× faster inference; (2) 250 real-world handover trials comparing our method with the baseline across 5 objects, 5 hand postures, and 5 angles, showing an improvement of 18.4 percentage points in success rate; (3) 450 trials under controlled adverse lighting (darkness and glare), where our dual-path perception method achieves 82.7% overall success, surpassing single-camera baselines by up to 39.4 percentage points; and (4) a comparative experiment against a state-of-the-art multimodal H2R handover method under identical adverse lighting, where our system achieves 75.0% success (15/20) versus the baseline’s 15.0% (3/20), further confirming the lighting robustness of our design. These results demonstrate the system’s robustness and generalization in challenging H2R handover scenarios.

## 1. Introduction

Human-to-robot (H2R) handovers, where a robot takes an object from a human hand, are a significant task in human–robot interaction (HRI) [1]. Implementing handovers in fields such as healthcare, construction, and services profoundly contributes to the natural collaboration between humans and robots. Recently, with the rapid development of humanoid robots, it is foreseeable that for a considerable period, these robots will assist humans in completing mundane, repetitive, physically demanding, and even high-risk tasks. Against this backdrop, H2R handovers are gaining unprecedented importance, while also facing more severe practical challenges. For instance, in structured industrial settings, robots operate under constant lighting conditions. However, in new HRI settings, robots must move around and engage in verbal communication and physical object transfer with humans. In these scenarios, variations in lighting conditions and the need for generalizing grasps for unknown objects and scenes for H2R handovers pose significant challenges to the smooth completion of these tasks. Therefore, during such handovers, robots need to have strong perception to handle the complexity and diversity of scenes, objects, and bodily movements.

Despite these challenges in H2R handovers, numerous studies have explored how to perceive unknown environments without prior knowledge. Most existing works rely on RGB image inputs for core perception. These methods can be broadly categorized into two streams: those employing RGB-based learning networks for object segmentation and localization [2,3,4,5,6], and those extracting human skeleton keypoints from RGB video streams to guide motion planning [7,8,9]. Although such approaches achieve high precision under stable indoor lighting, their perception performance degrades severely in dynamic lighting scenarios (e.g., glare or darkness), as RGB imaging is highly sensitive to illumination variations. Meanwhile, several works do not include explicit semantic segmentation to simplify the pipeline [10,11,12]. However, this design often leads to cascaded errors in downstream grasp generation, as it cannot provide accurate hand–object boundary information in H2R scenarios with frequent occlusion.

In response to the above limitations of RGB-based methods, some studies have leveraged the robustness of point cloud and depth data to mitigate illumination interference. Orsula et al. [13] used point cloud data for three-dimensional reconstruction and employed deep reinforcement learning to achieve effective grasping under the harsh lunar lighting. Li et al. [14] enhanced point cloud features with the angular information of normal vectors from corresponding depth maps, realizing RGB-independent 3D semantic segmentation and six degrees of freedom (6-DoF) object pose prediction. Ma et al. [15] explored the grasping of objects represented by multi-scale point clouds, applying 3D semantic segmentation to perceive cluttered scenes with multiple objects. Zhang et al. [16] distinguished between objects and human bodies during handovers using thermal sensors, creating a corresponding point cloud dataset and segmenting multimodal data, including point clouds, based on RandLA-Net in practical use. These advancements have demonstrated a certain robustness under complex lighting conditions. However, these algorithms either require sophisticated sensing equipment for additional data [16], or their generalization capabilities are insufficient to accommodate the complex shapes of human hands along with objects [14]. Crucially, the hand occlusion and unpredictable orientation changes in H2R handovers fundamentally limit the generalization of uncluttered-scene grasp methods [13,15] to such scenarios.

Recent advances in H2R handovers have explored diverse learning-based strategies to enhance interaction fluency and adaptability. Zhong et al. [17] encoded spatiotemporal features in a diffusion model to improve the smoothness of robot motion planning during handovers. van Zoelen et al. [18] formulated the decision process as a three-step Q-learning problem, enabling real-time adaptation based on human motion cues. Tulbure et al. [19] integrated semantic features from large language models (LLMs) to improve the precision of object part identification during task-oriented handovers. While these methods demonstrate the potential of feature fusion and policy learning, they do not explicitly account for the joint constraints of the robot arm and the geometric variations introduced by multi-angle human–robot relative positioning.

In summary, existing studies still exhibit three limitations:

1. Fragility of RGB-based perception, which degrades system robustness under adverse lighting conditions
2. Insufficient fusion of human–robot spatial relational features in existing 3D semantic segmentation methods
3. Inadequate posture and scenario adaptation in grasp generation approaches for H2R handover tasks

**Figure 1:**
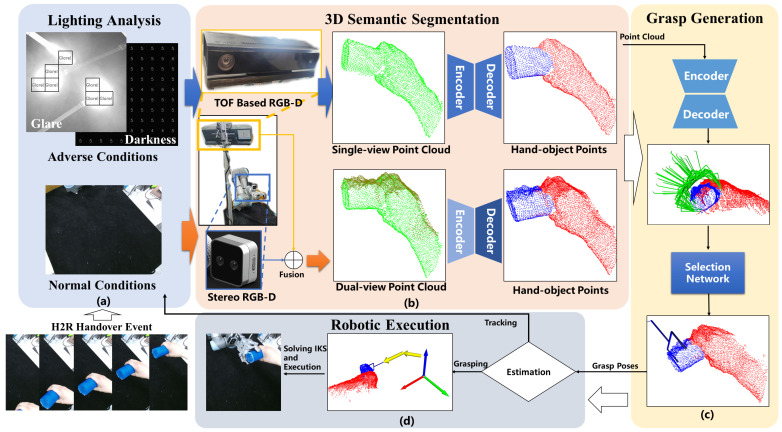
Overview of our proposed handover framework. (**a**) Analyzing lighting conditions to determine the visual source from two cameras. (**b**) Employing a semantic segmentation model for real-time 3D semantic segmentation of captured point clouds, identifying points belonging to the human hand (red) and the object (blue). (**c**) Utilizing a grasp generation model to predict the optimal 6-DoF grasping pose. (**d**) Tracking the object or executing the handover.

To address the aforementioned constraints, we present an H2R handover system with a dual-path perception pipeline. First, the system fuses perception data from a stereo RGB-D camera (eye-in-hand) and a ToF camera (fixed scene) under normal lighting, and switches to the ToF camera for reliable perception under glare and darkness. Subsequently, our 3D semantic segmentation algorithm is extended from Point Transformer v3 (PTv3) [20], which has demonstrated exceptional performance across over 20 indoor and outdoor point cloud tasks. Finally, a grasp generation algorithm adapted from GraspNet is proposed to select an optimal pose for handover. The framework of the system is illustrated in Figure 1.

More specifically, the main contributions of this article are listed as follows:

- A lighting-adaptive dual-path perception pipeline that dynamically switches between a stereo RGB-D camera and a time-of-flight (ToF) camera, ensuring reliable perception under both normal and adverse lighting conditions (glare and darkness).
- A geometry-aware 3D semantic segmentation model that extends Point Transformer v3 (PTv3) with a T-Net module and a self-attention mechanism to encode relative positional angles between human and robot, enabling accurate real-time segmentation of hand–object interactions from multiple viewpoints.
- A grasp generation framework adapted from GraspNet with a dedicated optimal grasp selection module that accounts for human hand postures and robot motion constraints, significantly improving grasp success in dynamic H2R scenarios compared with conventional object-centric grasp methods.

## 2. Related Work

### 2.1. Lighting-Robust Multimodal Perception

Existing research on lighting-robust perception can be categorized into two technical routes: one relies on specialized sensors to compensate for visual information under extreme lighting, while the other explores algorithm-level optimizations based on consumer-grade RGB-D cameras.

For the first route, researchers have introduced non-visual sensors and complementary imaging schemes to overcome the limitations of conventional cameras. Lin et al. [21] fused event cameras with conventional frame-based cameras, drawing inspiration from the human pupil control pathway under strong light, and proposed a peripheral vision transformation-based image processing method to achieve robust hand keypoint detection under glare. Qi et al. [22] developed a visual-tactile pre-training multimodal framework that uses tactile signals to compensate for visual degradation under extreme lighting, achieving dexterous manipulation under drastic illumination changes. However, these methods rely on specialized sensors (e.g., event cameras, tactile sensors, thermal sensors), which are less compatible with the most mature equipment used in industrial scenarios.

Given the limitations of specialized sensors, other studies have explored lighting-robust perception using consumer-grade depth cameras (e.g., structured light, ToF). Huang et al. [23] fused RGB semantic features with point cloud geometric data and proposed a joint pose optimization network that achieves stable grasp pose estimation under low-light conditions. Liu et al. [24] designed an early fusion strategy to jointly encode point cloud geometry and image texture features, improving the robustness of grasp detection under low illumination. However, most of these works focus on static grasping in low-light scenes and do not address the more common challenge of glare in industrial environments or the geometric constraints inherent in human–robot collaboration.

To address these limitations, we propose a lighting-robust dual-path perception framework based on the ToF modality. Unlike existing approaches that rely on specialized sensors or are limited to static grasping tasks, our method deploys a high real-time perception algorithm within a dual-path framework, achieving stable human–robot collaboration under both glare and darkness conditions.

### 2.2. Grasp Generation Methods in H2R Handover

Existing learning-based methods for H2R grasp generation can be broadly categorized into two groups: end-to-end 6-DoF grasp prediction and policy learning-based motion planning. For end-to-end 6-DoF grasp prediction, most H2R handover works in this line [3,5,11] build their grasp prediction backbone on pre-trained models from public general-purpose static grasp datasets, including ACRONYM [25], GraspNet [26], and DexYCB [27]. These datasets are primarily developed for unoccluded planar object grasping tasks, and do not explicitly model the inherent spatial constraints and diverse human–robot relative pose variations that are unique to H2R handover scenarios, leading to limited generalization across varied hand postures and relative positions in handover tasks. For policy learning-based methods, some studies use reinforcement learning to bypass explicit grasp pose prediction [4,10]; however, these approaches have limited generalization to novel objects and cannot guarantee consistent grasp performance in unstructured HRI scenarios. To address this generalization limitation in H2R handover scenarios, we extend GraspNet with a dedicated optimal grasp selection module optimized for handover tasks. The module explicitly accounts for constraints from hand postures, human–robot relative angles, and robot motion planning, improving grasp adaptability in H2R handover scenarios.

### 2.3. 3D Semantic Segmentation for H2R Scenarios

Point-based transformer models have become the mainstream for 3D point cloud semantic segmentation, among which Point Transformer v3 (PTv3) has achieved state-of-the-art (SOTA) performance on multiple indoor and outdoor benchmarks with its efficient sequential feature encoding capability [20]. However, H2R handover scenarios impose unique demands on segmentation models: the model must not only segment hand and object points under frequent occlusion, but also capture the relative spatial relationship between the human hand and robot, which directly determines the feasibility of the generated grasp. Existing general segmentation models lack targeted optimization for this spatial relationship feature, leading to poor generalization under multi-angle handover configurations.

Therefore, we extend the PTv3 backbone by integrating a T-Net module and an angle-aware self-attention mechanism, which effectively fuses the relative positional angular features between human and robot. This design enables the model to maintain high segmentation accuracy under variable viewpoints and hand posture changes in H2R handovers.

## 3. Framework of the Proposed Handover System

The workflow of the entire system comprises four stages: Lighting Analysis, Object Perception, Grasp Generation, and Execution, corresponding to (a), (b), (c), and (d) in Figure 1, respectively.

### 3.1. Lighting Analysis

The lighting analysis stage evaluates lighting conditions using RGB data. This part functions to distinguish normal lighting from disruptive conditions such as darkness and glare, ensuring our RGB-independent method activates appropriately. Figure 1a illustrates RGB image frames under these respective conditions.

The analysis is implemented using threshold filtering. First, the captured RGB image is divided into a 10×10 grid. Each grid region is then converted to a single-channel image. During system operation, we continuously compute the average pixel value for each region.

For glare detection, we employ a concentric-layer verification strategy: *(1)* We first identify candidate glare regions where the average intensity exceeds 240; *(2)* For each candidate region, we calculate the average intensities in concentric square regions at radial distances of 1, 3, and 5 layers outward from the center; *(3)* The verification requires that these outer-layer averages follow a specific attenuation pattern: >140 for layer 1, >80 for layer 3, and >50 for layer 5. This multi-layer verification helps distinguish true glare incidents from white objects, as demonstrated in the upper portion of Figure 1a.

For low-light condition detection, we apply a global intensity threshold. A low-light condition is flagged when the maximum average intensity among all regions falls below 50.

Based on the aforementioned principles, this module determines in real time whether the lighting condition is adverse for RGB-based perception (i.e., darkness or glare). Under adverse lighting conditions, the system employs the ToF-based perception modality; under normal conditions, it fuses the dual-camera perception channels. Such adaptive switching guarantees robust perception against lighting variations.

### 3.2. 3D Semantic Segmentation

The 3D semantic segmentation stage segments real-time point clouds into three categories: hands, objects, and other, as illustrated in Figure 1b.

#### 3.2.1. Algorithm Architecture

**Figure 2:**
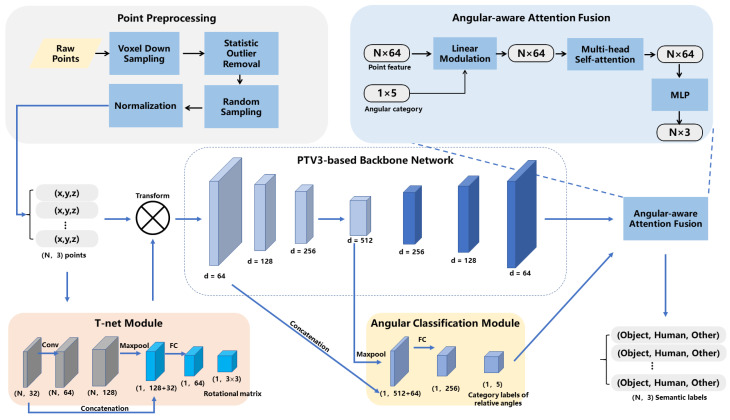
Architecture of our point cloud segmentation network. It employs a PTv3 backbone, augmented with a T-Net and an angular feature fusion module.

The proposed segmentation algorithm is based on a deep learning model utilizing PTv3 as the backbone network, augmented with T-Net and relative angle feature fusion. The model architecture is depicted in Figure 2.

Initially, the raw point cloud, containing approximately 10,000 to 30,000 points, undergoes voxel downsampling and statistical filtering, reducing the point count to about 2000 to 5000. These are then randomly sampled down to a fixed number of 2000 points, serving as the input to our model.

Subsequently, a T-Net module is applied to predict a 3×3 affine transformation matrix for the input point cloud, achieving spatial alignment of point clouds across different viewpoints. The T-Net follows the classic design from PointNet [28] with a tailored cross-layer feature fusion strategy, which retains shallow feature information via residual-like concatenation of multi-scale global features. It first employs three shared multi-layer perceptron (MLP) layers (output dimensions 32, 64, and 128) for point-wise feature extraction, with the 32-dimensional output of the first MLP layer preserved. After obtaining the 128-dimensional point-wise features, a max pooling layer is applied to generate a 128-dimensional global shape feature. Meanwhile, the preserved 32-dimensional point-wise features are aggregated into a 32-dimensional global feature via global max pooling. These two global features are concatenated along the channel dimension to form a 160-dimensional fused global feature, which is directly fed into two fully connected layers (output dimensions 64 and 9) to regress the 3×3 transformation matrix. A batch normalization layer and ReLU activation are applied after each linear layer, except the final output layer.

Having completed spatial alignment of the input point cloud with the T-Net module, we next introduce a relative angular information fusion branch tailored for H2R handover scenarios, beginning with the definition of scenario-adapted angular categories.

**Figure 3:**
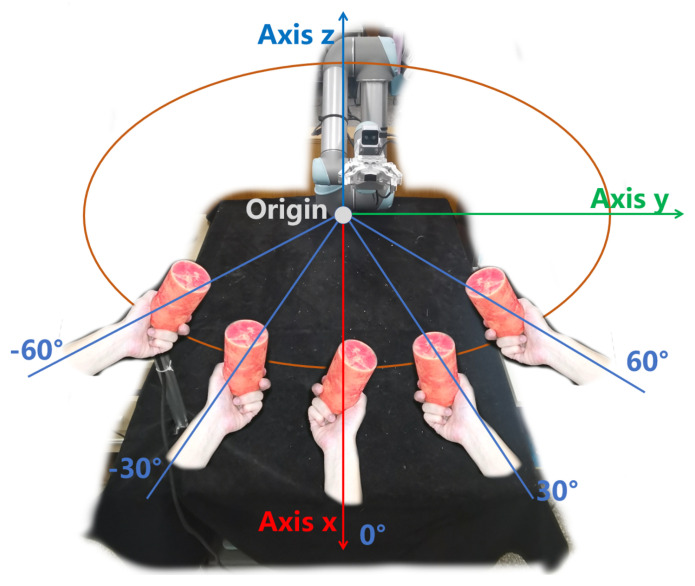
Definitions of five relative angular categories, determined by the relative positional angles between the human hand and the robot base.

A protocol of five relative angular categories is designed based on the practical constraints of H2R handover scenarios: (1) The reachable range of the UR5 robotic arm used in our system, where relative angles beyond ±60° will lead to the robot end-effector exceeding the optimal operation range and increasing the difficulty of motion planning; (2) The natural interaction habits of humans in handover tasks, where handovers within ±60° relative to the robot base account for most of the daily handover scenarios, verified by our pre-experiments of human handover behavior. We select an interval of 30° to balance the angular resolution and the complexity of the classification task, finally defining 5 categories: 0°, ±30°, and ±60°, covering the common reachable range of human–robot handovers in our scenario (as depicted in Figure 3).

Finally, to integrate the relative angular information between the human hand and the robot into the dense semantic segmentation task, we design two cascaded functional modules: an angular classification module for global relative angle prediction, and an angular-aware attention fusion module for feature-level angular information injection. The angular classification module takes multi-scale features from the PTv3 encoder as input: it concatenates the 512-dimensional global feature output by the final PTv3 encoder stage, with the 64-dimensional point-wise feature from the first downsampling stage of PTv3 (after global max pooling to aggregate into a global feature vector). It then maps this concatenated multi-scale feature to the five angle category predictions via two fully connected layers (output dimensions 256 and 5) with batch normalization and ReLU activation. For the downstream angular-aware attention fusion module, we first obtain the five-dimensional one-hot encoded vector $v_{\mathrm{ang}}\in {\left\{0,1\right\}}^{5}$ by discretizing the predicted angle category probability distribution ${\hat{y}}^{\mathrm{ang}}$ from the aforementioned angular classification module. We map this one-hot vector to a 64-dimensional angle embedding vector $f_{\mathrm{angle}}$ via a learnable embedding layer, where the embedding weights are updated synchronously with the network during training. Then, we perform linear modulation on the final upsampled per-point features (64-dimensional) from the PTv3 decoder to inject the angular information:

(1) $$F_{\mathrm{mod}}=F_{\mathrm{point}}⊙\mathrm{MLP}\left(f_{\mathrm{angle}}\right)$$

where $F_{\mathrm{point}}\in R^{N\times 64}$ is the per-point feature output by the PTv3 decoder (*N* is the number of input points), $f_{\mathrm{angle}}\in R^{64}$ is the learnable angle embedding vector, and MLP is a 2-layer perceptron that maps the 64-dimensional angle embedding to a 64-dimensional weight vector with the same dimension as the per-point feature. The operator ⊙ denotes the Hadamard (element-wise) product, where the weight vector is broadcast along the point dimension to match the shape of $F_{\mathrm{point}}$ before multiplication. The modulated feature $F_{\mathrm{mod}}$ are then fed into a 4-head multi-head self-attention (MHSA) layer to capture the long-range dependencies between hand and object points, followed by an MLP layer to output the final per-point semantic category predictions.

#### 3.2.2. Objective Function

The optimization objective of the segmentation network comprises two task-aligned components: an angular classification loss and a point cloud segmentation loss. To avoid feature learning conflicts between the global angle classification task and the dense point-wise segmentation task, we adopt a two-stage training strategy for this network, where the two loss components are activated sequentially across different training stages.

During the first training stage, we exclude the native upsampling decoder of PTv3, retain its downsampling and encoding backbone to output high-dimensional global features, and only pre-train an angular classification module via fully connected layers. This stage aims to let the PTv3 backbone learn to extract angle-aware global features for H2R handover scenarios, without involving any semantic segmentation task. The loss function for this stage is defined as the mean angular classification loss over a batch of point clouds:

(2) $$L_{\mathrm{stage}1}=L_{\mathrm{ang}}=-\frac{1}{N_{\mathrm{sample}}}\sum_{i=1}^{N_{\mathrm{sample}}}\sum_{c=1}^{5}y_{i,c}^{\mathrm{ang}}\log\left({\hat{y}}_{i,c}^{\mathrm{ang}}\right)$$

where $N_{\mathrm{sample}}$ denotes the number of point cloud samples in the batch, 5 is the number of angle categories, $y^{\mathrm{ang}}\in {\left\{0,1\right\}}^{5}$ is the one-hot vector of the ground-truth angle category for the *i*-th point cloud, and ${\hat{y}}^{\mathrm{ang}}$ represents the predicted probability distribution over angle categories for the *i*-th point cloud.

In the second stage, we restore the complete PTv3 encoder–decoder architecture and initialize the network with weights pre-trained during stage 1. The stage 2 objective integrates angular information through attention mechanisms and combines two loss components, with the angular classification module fine-tuned synchronously:

(3) $$\begin{array}{c} L_{\mathrm{seg}}=-\frac{1}{N_{\mathrm{point}}}\sum_{i=1}^{N_{\mathrm{point}}}\sum_{c=1}^{C}p_{i,c}^{\mathrm{seg}}\log\left({\hat{p}}_{i,c}^{\mathrm{seg}}\right)\\ L_{\mathrm{stage}2}=\alpha L_{\mathrm{ang}}+\beta L_{\mathrm{seg}} \end{array}$$

Here, $L_{\mathrm{ang}}$ is computed identically to Equation (2). $L_{\mathrm{seg}}$ denotes the mean point-wise segmentation loss, where $N_{\mathrm{point}}$ is the total number of points across all point clouds in the batch, *C* is the number of point-wise semantic categories, $p^{\mathrm{seg}}\in {\left\{0,1\right\}}^{C}$ is the one-hot vector of the ground-truth semantic category for the *i*-th point, and ${\hat{p}}^{\mathrm{seg}}$ represents the predicted probability distribution over semantic categories for the *i*-th point. The coefficients α=0.5 and β=0.5 balance the contributions of the two loss components.

#### 3.2.3. Semantic Dataset Construction

**Figure 4:**
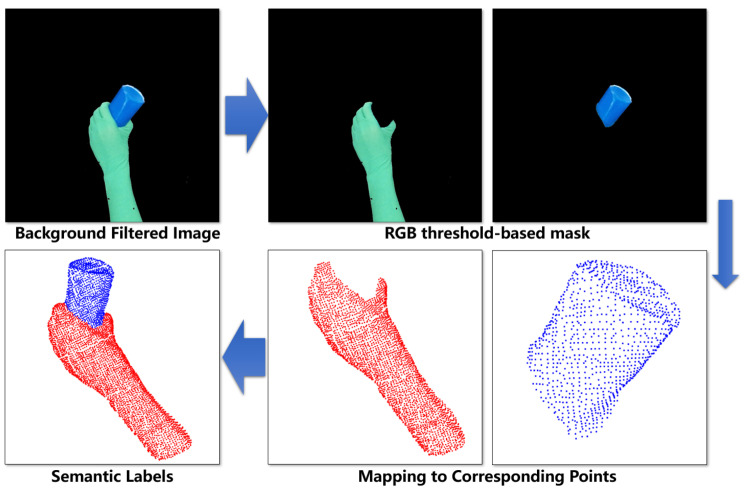
Points data collection pipeline for semantic segmentation. Leveraging RGB filtering, volunteers wore green gloves during handovers, enabling automatic generation of image masks. This facilitated automatic annotation of semantic labels.

We annotate point clouds using captured depth images of human hands carrying objects. As shown in Figure 4, data collection involves volunteers wearing green gloves holding diverse objects while performing continuous handover-like motions.

We apply RGB thresholding to extract distinct pixel masks for hands and objects. Then, hand–object points are categorized using pixel-to-point mapping for each depth frame. This approach efficiently captures: (1) Varied handover postures (2) Diverse object geometries (3) Natural motion trajectories (4) Relative angles between human and robot.

**Figure 5:**
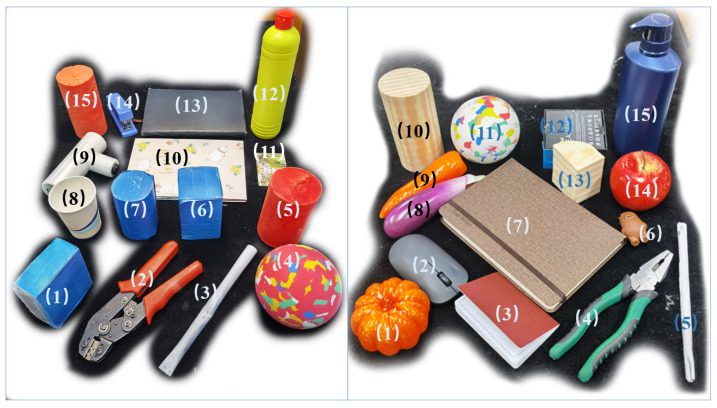
Training objects: (1) block, (2) plier, (3) rod, (4) sphere, (5) cylinder a, (6) quadrangular, (7) cylinder b, (8) paper cup, (9) massager, (10) mirror, (11) credit card, (12) bottle, (13) notebook, (14) stapler, (15) cylinder c. Testing objects: (1) pumpkin, (2) mouse, (3) license, (4) plier, (5) rod, (6) pendant, (7) book, (8) eggplant, (9) carrot, (10) cylinder, (11) sphere, (12) box, (13) prism, (14) apple, (15) bottle.

The dataset includes 15 object categories covering everyday items and geometric primitives (Figure 5, left). After rigorous cleaning of missegmented points, we randomly add background disturbance points from the robotic arm and camera mounting rack to the annotated dataset to improve the model’s robustness to scene clutter. The final dataset contains 7500 handover point clouds with: (1) Per-point semantic labels (hand/object/other) (2) Instance-level angular annotations (0°/±30°/±60°).

### 3.3. Grasp Generation

**Figure 6:**
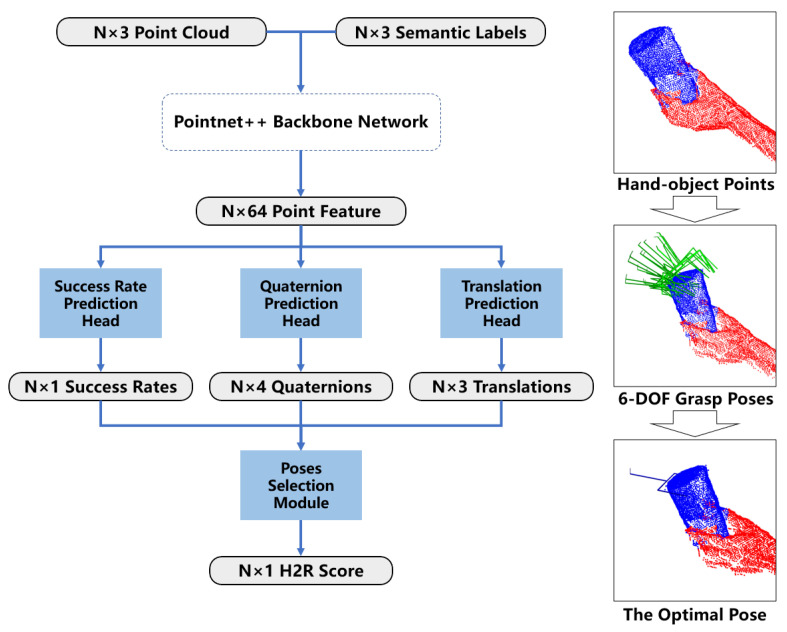
Architecture of the grasp generation network. A PointNet++ backbone extracts point-wise features, processed by three prediction heads to generate grasp candidates. A selection module then predicts the optimal 6-DoF pose.

The grasp generation module (Figure 1c) predicts the real-time optimal grasp pose using the segmented points obtained in Section 3.2. The algorithm consists of a poses generation module (Figure 6 middle right) and a pose selection module (Figure 6 lower right). Depending on lighting conditions, the system either uses the dual-camera (eye-in-hand and eye-on-scene cameras) point category labels or the single-camera (eye-on-scene camera only) point category labels as input to determine the optimal grasp.

#### 3.3.1. Grasping Dataset Construction

We construct a grasping dataset consisting of simulation and real-world data, where the simulation data is used for grasp pose learning, and the real-world data is used for optimal grasp selection. Both parts are composed of single-view point clouds of daily objects, with task-specific annotation designs tailored to different training objectives.

**Figure 7:**
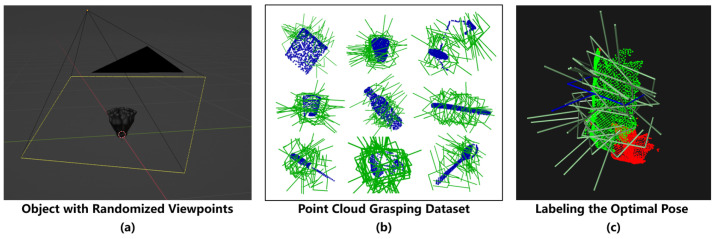
Grasp data acquisition and annotation pipeline: (**a**) Generating diverse single-view and multi-angle point clouds of objects using Blender. (**b**) Grasp poses from the ACRONYM dataset [25]. (**c**) Manual selection of optimal grasp pose indices on real handover point clouds.

The simulation dataset contains 12,000 sets of single-view point clouds, generated from 30 daily objects selected from the ACRONYM dataset [25]. For each object, we adopt the complete 6-DoF feasible grasp pose annotations provided by ACRONYM, and use Blender to simulate single-view point clouds under different relative camera poses, as shown in Figure 7a,b. This dataset provides large-scale, high-precision annotations of geometrically feasible grasps and is used for the pre-training of the grasp pose generation module. It should be noted that this dataset only provides annotations of all feasible grasps for each object, without global optimal grasp labels.

The real-world dataset contains 3000 sets of dual-view point clouds, captured by the dual-camera system in the actual deployment scenario. Object point clouds are obtained from raw data via the semantic labeling method described in Section 3.2. For each set of object point clouds (including those captured by the eye-in-hand and scene cameras), we manually annotate a unique global optimal 6-DoF grasp pose. The two point clouds in the same set share an identical optimal grasp annotation, to ensure that each camera can independently and effectively predict the optimal grasp. The annotation criterion is to minimize the relative angle between the end-effector and the robot base under the premise of collision-free and valid grasping, so as to improve the execution efficiency of the robot in real-world scenarios, as shown in Figure 7c. This dataset provides scenario-specific optimal grasp annotations for the target H2R system and is used for the training of the grasp selection module.

Through the above targeted annotation design, the complete grasping dataset includes 15,000 sets of point clouds, covering the daily objects used in the actual deployment. Examples of the real objects are listed on the left side of Figure 5.

#### 3.3.2. Algorithm Architecture

The network architecture is depicted in Figure 6. The H2R optimal grasp pose is determined from the cameras’ single-view point cloud input. First, a PointNet++ backbone network extracts point-wise features. Then, point-wise grasp poses are predicted using three prediction heads, which generate success rates, quaternions, and translations for the grasp poses. Finally, the optimal grasp pose is selected for each depth frame using a pose selection module. Both the prediction heads and the selection module are implemented using Pointnet-like structures followed by fully connected layers.

#### 3.3.3. Grasp Representation and Objective Function

In the point decoding process, each grasp G is represented as:

(4) $$G=\left[c,q,t\right],$$

where c∈R denotes the probability of grasp success, $q\in R^{4}$ is the rotation quaternion, and $t\in R^{3}$ is the gripper center translation, defining a 6-DOF end-effector pose.

The input point cloud is normalized by centering at the origin and scaling to a unit sphere. For each point $p_{i}$, we identify *m* annotated grasps $G_{j}^{*}$ within a radius of 0.05 units based on their $t_{j}^{*}$. The target success label is:

(5) $$c_{i}^{*}=\left\{\begin{array}{cc} 1 & \mathrm{if}\;\sum_{j=1}^{m}I_{\mathrm{success}}\left(j\right)\geq 1\\ 0 & \mathrm{otherwise}. \end{array}\right.$$

(1) Learning from Point-Wise Poses

For a batch of *B* points, we compute:

- Confidence Loss (Binary cross-entropy):

(6) $$L_{c}=\frac{1}{B}\sum_{i=1}^{B}\left\{\begin{array}{cc} -\log(c_{i}) & \mathrm{if}\;c_{i}^{*}=1\\ -\log(1-c_{i}) & \mathrm{otherwise} \end{array}\right.$$

- Pose Loss (Mean Squared Error):

(7) $$\begin{array}{c} L_{q}=\frac{1}{B}\sum_{i=1}^{B}\min_{j\in N_{i}}{∥q_{i}-q_{j}^{*}∥}_{2}^{2}\\ L_{t}=\frac{1}{B}\sum_{i=1}^{B}\min_{j\in N_{i}}{∥t_{i}-t_{j}^{*}∥}_{2}^{2} \end{array}$$

where $N_{i}$ denotes the set of ground-truth grasps near $p_{i}$.

(2) Learning from the Optimal Pose

An optimal pose selection module selects top-*K* grasps from *N* predicted candidates ${\left\{G_{i}\right\}}_{i=1}^{N}$ using the object point cloud. For each candidate:

(8) $$D_{i}=\min_{j}\left({∥q_{i}-q_{j}^{*}∥}_{2}+{∥t_{i}-t_{j}^{*}∥}_{2}\right)$$

Define binary selection targets as:

(9) $$s_{i}^{*}=\left\{\begin{array}{cc} 1 & \mathrm{if}\;G_{i}\in \mathrm{top}-K\;\mathrm{ranked}\;\mathrm{by}\;D_{i}\\ 0 & \mathrm{otherwise} \end{array}\right.$$

The selection loss is:

(10) $$L_{s}=\frac{1}{N}\sum_{i=1}^{N}\left\{\begin{array}{cc} -\log(s_{i}) & \mathrm{if}\;s_{i}^{*}=1\\ -\log(1-s_{i}) & \mathrm{otherwise} \end{array}\right.$$

where $s_{i}$ is the predicted selection score.

(3) Training Strategy Implementation

The combined loss integrates all components:

(11) $$L_{\mathrm{total}}=\alpha L_{c}+\beta \left(L_{q}+L_{t}\right)+\gamma L_{s}$$

To address the domain gap between simulation and real-world data, as well as the mismatch of annotation dimensions between the two datasets, we adopt a two-stage training strategy based on the proposed total loss function, with stage-specific hyperparameter settings and gradient control:

- **Stage 1**: This stage uses only the simulation dataset with complete 6-DoF grasp pose annotations, aiming to learn the general ability of feasible grasp pose generation. In this stage, we set the weight of the selection loss γ=0 to disable the contribution of $L_{s}$ to the total loss, and freeze the weights of the pose selection module to stop its gradient update. Only the PointNet++ backbone and the three grasp prediction heads are optimized, with the loss weight set to α=0.5, β=1.0. This setting balances the learning of grasp success, confidence, and pose regression accuracy.
- **Stage 2**: This stage uses only the real-world dataset with manually annotated optimal grasp poses, aiming to align the model with the real deployment scenario and the custom optimal grasp criterion. In this stage, we set the weights of the confidence loss and pose loss α=0, β=0 to disable the contribution of $L_{c}$, $L_{q}$ and $L_{t}$ to the total loss, and freeze the weights of the pre-trained PointNet++ backbone and grasp prediction heads. Only the pose selection module is optimized, with the loss weight set to γ=1.0, to learn the scoring of optimal grasps without damaging the pre-trained pose generation ability.

### 3.4. Robot Execution and Control

The robot execution process is initiated upon detection of object point clouds from either camera. Based on continuous measurements of the Euclidean distance changes in the object’s point cloud, the system makes decisions on whether to maintain a fixed distance and track the object or to initiate the grasp. As illustrated in Figure 1d.

The arm’s joint angles are calculated using inverse kinematics, subject to constraints on the joint ranges to ensure safe motion. Upon grasp execution, the robot arm follows a Cartesian trajectory. It first moves to a pre-grasp position, offset 10 centimeters backward from the grasp pose along its approach direction. It then moves 10 centimeters forward to the target grasp position and closes the gripper. Subsequently, the grasping result is evaluated based on the torque and angle readings obtained from the gripper controller. The gripper control is torque-based. We employ a closing force of 50 Newtons to close the gripper, ensuring a stable yet safe grip on the test objects without causing damage.

## 4. Experimental Setup and Evaluation Protocol

**Figure 8:**
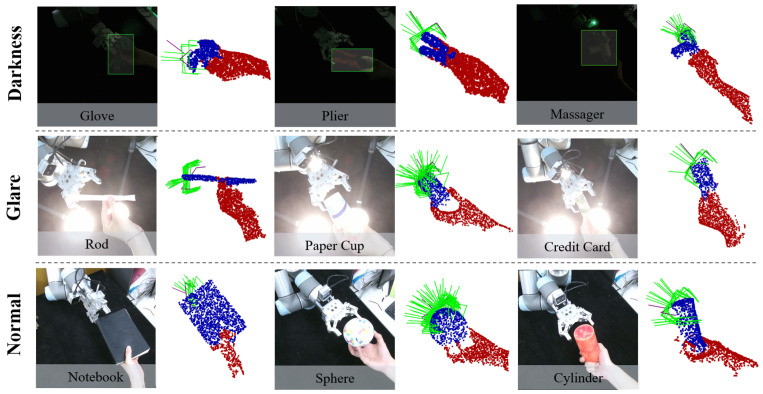
Adapting to diverse lighting conditions, our handover system successfully handles multiple objects delivered by humans.

Figure 8 provides an overview of the proposed handover system, demonstrating its ability to handle multiple objects under various lighting conditions. The following subsections detail the system setup, datasets, and experimental protocols.

### 4.1. System Setup

The robotic system uses a UR5 arm (Universal Robots, Odense, Denmark) equipped with a two-finger parallel gripper (Nonead, Suzhou, China) at the end-effector. The arm and the gripper are controlled by an Ubuntu computer running ROS Noetic. Machine learning algorithms run on a computer with an RTX 2080ti GPU, utilizing PyTorch 1.12.1 and Python 3.9, to issue position control commands. Communications between all devices are facilitated through a network switch.

Perception is provided by a KinectV2 ToF-based RGB-D camera (Microsoft, Redmond, WA, USA) serving as the fixed scene camera, and a Realsense D405 RGB-D camera (Intel, Santa Clara, CA, USA) serving as the eye-in-hand camera.

Depth information: The scene camera operates on the ToF principle with active near-infrared projection, enabling valid depth acquisition under the adverse lighting conditions constructed in the experiment. The eye-in-hand camera is based on passive near-infrared (NIR) stereo vision, whose depth perception performance degrades significantly under insufficient illumination or overexposure.

RGB information: Both cameras are equipped with an independent RGB imaging channel to provide color image data for the perception pipeline.

The experiment received approval from the Ethics Committee of Southeast University (2020-SR-362).

### 4.2. Environmental Lighting Setup

Three controlled lighting scenarios were constructed for all handover experiments, with illuminance measured by a AS803 calibrated digital illuminance meter (Xima, Dongguan, China) at the camera lens position to ensure replicability:

- **Normal lighting**: Standard ambient laboratory lighting, with a measured illuminance of 350±50 lux at the camera lens, where both cameras maintained full effective point cloud acquisition performance.
- **Dark environment**: Established using full blackout curtains and all ambient lights turned off, with a measured illuminance of <3 lux at the camera lens. Under this condition, the effective point cloud acquisition rate of the passive NIR stereo camera dropped below 10%, while the active ToF camera maintained stable depth imaging.
- **Glare interference**: Generated by directional 32 W LED arrays (220 V, 12 W + 12 W + 8 W configuration) directed at the core perception camera, with a measured illuminance of >930 lux at the camera lens. Under this condition, the effective point cloud acquisition rate of the passive NIR stereo camera dropped below 40% due to overexposure, while the ToF camera retained stable imaging performance.

### 4.3. Dataset Train/Test Split

This work constructs two dedicated datasets for semantic segmentation and grasp generation, respectively.

#### 4.3.1. Semantic Segmentation Dataset

The dataset contains 7500 annotated handover point cloud samples for training, covering 15 daily objects and 5 relative positional angle categories (0°,±30°,±60°). We adopt an object-level train/test split to rigorously validate the model’s generalization ability: another 15 unseen novel objects are used for testing (50 samples per object, total 750 test samples). A detailed data annotation pipeline is described in Section 3.2.3.

#### 4.3.2. Grasp Generation Dataset

The dataset consists of 12,000 simulation samples and 3000 real-world samples for training and testing, with a 9:1 object-level train/test split. Simulation data is generated from the ACRONYM dataset via Blender, and real data is collected from the same handover scenarios as the segmentation dataset. Detailed data construction is described in Section 3.3.1. Notably, simulation environment is only used for constructing the grasp generation data, and all handover experiments (Section 5.2, Section 5.3 and Section 5.4) in this work are conducted in real physical environments.

### 4.4. Evaluation Metrics

We adopt the following metrics to quantitatively evaluate the performance of the proposed system and baseline methods:

#### 4.4.1. Semantic Segmentation Metrics

- **mean Intersection over Union (mIoU)**: The primary metric for 3D semantic segmentation performance, calculated as the average Intersection over Union (IoU) across all semantic categories (hand, object, background). The IoU for a single category is defined as:
  $$\mathrm{IoU}=\frac{TP}{TP+FP+FN}$$
  where TP (True Positive) is the number of correctly classified points, FP (False Positive) is the number of incorrectly classified positive points, and FN (False Negative) is the number of missed positive points.
- **mean Accuracy (mAcc)**: The average classification accuracy across all semantic categories, calculated as the ratio of correctly classified points to the total number of points in each category, averaged over all categories.
- **Test Loss**: The cross-entropy loss of the semantic segmentation model on the test set, reflecting the convergence and generalization performance of the model.
- **Angle Classification Accuracy**: The ratio of correctly predicted relative human–robot angle categories to the total number of samples, reflecting the effectiveness of the angular feature fusion module.
- **Inference Latency**: The average time taken for a single point cloud frame to complete preprocessing, model inference, and postprocessing, in milliseconds (ms), reflecting the real-time performance of the algorithm.

#### 4.4.2. Grasp and Handover Performance Metrics

This metric is the core quantitative indicator for evaluating the end-to-end performance of the proposed human-to-robot (H2R) handover system, which comprehensively reflects the collaborative robustness of the perception module, grasp planning module, and robot motion control module. The handover success rate is calculated as:

$$\mathrm{Handover}\;\mathrm{Success}\;\mathrm{Rate}=\frac{\mathrm{Number}\;\mathrm{of}\;\mathrm{Successful}\;\mathrm{Handover}\;\mathrm{Trials}}{\mathrm{Total}\;\mathrm{Number}\;\mathrm{of}\;\mathrm{Valid}\;\mathrm{Handover}\;\mathrm{Trials}}\times 100\%$$

A handover trial is recorded as successful if and only if all of the following conditions are satisfied simultaneously:

1. No collision occurs between the robot (including the end-effector and arm body) and the human hand/body during the entire handover process;
2. The subject holds the object within the effective field of view (FoV) of the perception system and maintains a relatively static state. The start point of this static state is determined by the chamfer distance between the point clouds of two consecutive frames being less than 0.01 m, and this static state must persist for more than 3 s. During this 3-s static window, the system must output a valid 6-DoF grasp pose and initiate the execution;
3. The robot completes the planned grasp motion, and the gripper maintains a stable hold on the object for more than 3 s after grasping the object, with no slipping, dropping, or significant displacement of the object relative to the gripper.

A handover trial is recorded as failed if any of the following conditions occur:

- Collision between the robot and the human hand/body or the surrounding environment during the handover process;
- Motion planning failure of the robotic arm for the predicted target grasp pose (no collision-free executable trajectory can be generated within the joint constraints);
- No valid grasp pose is output by the system within the 3-s static window defined in condition (2) of the success criteria;
- The object slips, drops, or is detached from the gripper within 3 s after the grasping action is completed.

### 4.5. Statistical Analysis

All statistical analyses were performed using Python 3.9 (SciPy 1.11.0 library), with a significance level set to α=0.05 for all statistical tests. The analysis pipeline is as follows:

#### 4.5.1. Continuous Numerical Variables

For continuous measurement data, the analysis pipeline is as follows:

1. **Normality Test**: The Shapiro–Wilk test was used to verify whether the experimental data follows a Gaussian (normal) distribution.
2. **Statistical Description**: For data conforming to the normal distribution, results are reported as **mean ± standard deviation (SD)**; for data that does not conform to the normal distribution, results are reported as **median (interquartile range, IQR)**.
3. **Significance Test**: For comparisons between two independent groups, the independent samples *t*-test (for normally distributed data) or Mann–Whitney U test (for non-normally distributed data) was used. For comparisons among multiple groups, one-way ANOVA with Tukey’s post hoc test was applied to normally distributed data, and the Kruskal–Wallis H test was used for non-normally distributed data.

#### 4.5.2. Categorical Count Data

For categorical data (e.g., handover success/failure, classification of failure types), the analysis pipeline is as follows:

1. **Statistical Description**: Results are reported as **count (percentage, %)**, with the number of valid trials as the denominator.
2. **Significance Test**: The chi-square ($\chi^{2}$) test was used for inter-group comparison of success rates; Fisher’s exact test was used instead when the expected frequency of any cell in the contingency table was less than 5, to ensure the robustness of the test results.

## 5. Experimental Results and Discussion

### 5.1. Comparative Analysis in Semantic Segmentation Models

#### 5.1.1. Baselines

We compared the performances of various models on H2R 3D semantic segmentation. The models covered in our experiments include Swin3D-L [29], SphereFormer [30], Pointnet++ [28], PTv3 [20]. As of the time of writing, Swin3D-L ranks third on the S3DIS dataset, SphereFormer is third on SemanticKITTI, PTv3 leads on both SemanticKITTI and S3DIS, and Pointnet++ remains the most commonly adopted model in the field of robotics.

#### 5.1.2. Algorithmic Setup

Each model was trained for 10 independent trials, with each trial comprising 50 epochs, activated by a softmax function in the final layer. The input point clouds are normalized by translating them to the origin and scaling them to fit inside a unit sphere. Training is based on the Adam optimizer and a cross-entropy loss function. The learning rate is set to $1\times 10^{-3}$ at epoch 1, $1\times 10^{-4}$ at epoch 10, and $1\times 10^{-5}$ at epoch 20, respectively. A dropout rate of 0.3 is implemented.

All baselines follow their original official configurations. PointNet++ [28] uses its native encoder–decoder architecture. The PTv3 baseline [20] uses the native encoder–decoder architecture without the angular feature fusion module or two-stage training. SphereFormer [30] retains the original U-Net encoder–decoder structure with SparseConv. Swin3D-L [29] follows the original setup, with stage-wise feature channels of 80, 160, 320, 640, 640 and attention heads of 10, 10, 20, 40, 40.

Our proposed network adopts the two-stage training strategy described in Section 3.2.2, with core parameters aligned to the unified settings for fair comparison. In the pre-training stage, we remove the PTv3 decoder and train the PTv3 encoder with the angular classification module for 30 epochs, using the same optimizer and learning rate schedule. In the full training stage, we restore the complete PTv3 encoder–decoder architecture, initialize the encoder with pre-trained weights, and train the full network for 50 epochs (consistent with all baselines). The learning rate schedule remains unchanged, the angular classification module and decoder are updated synchronously, and loss weights are set to α=0.5, β=0.5 as defined in Section 3.2.2.

#### 5.1.3. Results

**Figure 9:**
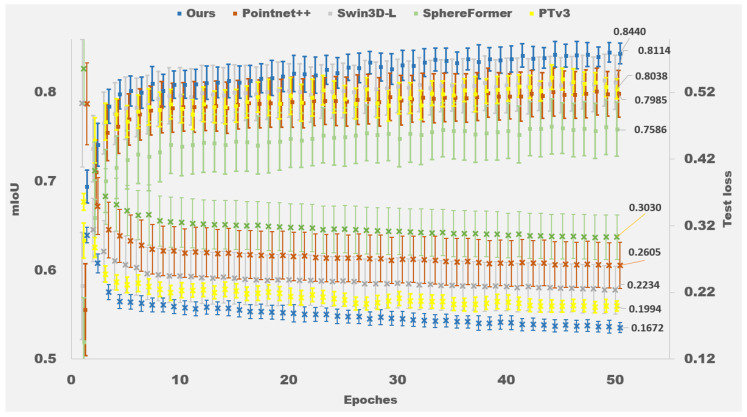
The mIoU and test loss curves of various models on our semantic segmentation dataset, where the markers represent the mean, and the error bars represent a 90% confidence interval.

The mIoU score for each epoch was recorded as a performance metric. The performances of various models are shown in Table 1, in which the mIoU curves are depicted in Figure 9. Our approach demonstrates clear superiority across all metrics: achieving the highest mIoU (84.4%), mAcc (90.3%), and lowest test loss (0.167) while maintaining competitive inference latency (78 ms). Notably, our model shows a 4.0% absolute improvement in mIoU over the baseline PTv3 architecture. The quantitative advantage is further visualized in Figure 9, where our method converges faster with tighter confidence intervals, indicating superior training stability.

All results are reported as mean ± standard deviation across 10 independent trials. The Shapiro–Wilk test indicated that the mIoU scores for each model were approximately normally distributed (p>0.05). One-way analysis of variance (ANOVA) revealed a significant difference among the models (p<0.001). Post hoc comparisons using Tukey’s HSD test indicated that our method achieved significantly higher mIoU than all baseline models (p<0.05 for each pairwise comparison). Similar statistical significance was observed for mAcc and test loss metrics.

#### 5.1.4. Ablation Study

**Figure 10:**
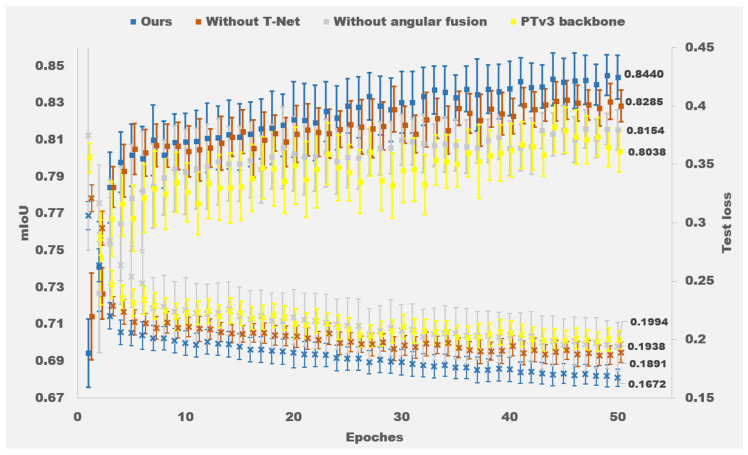
Ablation studies of the proposed segmentation model in four configurations: PTv3 backbone, without angular fusion, without T-Net, and full-structure.

We comprehensively evaluate T-Net and angular feature fusion as two components in the ablation study. The four configurations in the experiment are as follows: *(1)* PTv3 backboned, *(2)* Without angular fusion, *(3)* Without T-Net, and *(4)* Full structure (Ours). With experimental results being depicted in Table 2 and Figure 10, key observations reveal:

(1) Angular Fusion Contribution

Adding the angular fusion module alone (comparing Backboned to w/o T-Net) improves the mIoU for the ±30° category from 80.8% to 84.1% (a gain of 3.3%) and for the ±60° category from 77.7% to 79.7% (a gain of 2.0%). These gains validate that angular features are critical in visual perception for multi-positional H2R handovers.

(2) T-Net Contribution

Adding T-Net alone (comparing Backboned to w/o Angular) improves the mIoU for Human from 81.7% to 83.1% (a gain of 1.4%) and for Objects from 75.3% to 76.8% (a gain of 1.5%), demonstrating its efficacy in spatial feature alignment.

(3) Synergistic Effect

The full model achieves 84.4% mIoU, outperforming the backbone by 4.0% (from 80.4% to 84.4%), the configuration without angular fusion by 2.9% (from 81.5% to 84.4%), and the configuration without T-Net by 1.5% (from 82.9% to 84.4%). This confirms the complementary benefits of both components.

Complementary results in Table 3 confirm the angular classifier’s effectiveness, achieving 90.3% overall accuracy with best performance at 0° (94.8%). This demonstrates the robustness of the proposed method in encoding relative angle relationships between the human and the robot. Note that the “relative angle” entries in Table 2 are categorized based on the actual angle at which each test sample was captured, independent of whether the model incorporates an angular fusion module.

Statistical analysis (one-way ANOVA with Tukey’s HSD) on the mean mIoU of the four configurations showed that the full model significantly outperformed all ablated versions (p<0.05 for each pairwise comparison), confirming the contribution of each component. The angular classifier accuracy in Table 3 is also reported as mean ± standard deviation, serving as a descriptive performance metric.

#### 5.1.5. Discussion

Our comprehensive evaluation establishes three key insights:

First, the specialized nature of H2R handover scenarios, characterized by positionally concentrated point clouds and small class diversity (Human, Objects, Others), fundamentally shapes model performance. While the limited categories (3 classes vs. typical 20+ in datasets like S3DIS) appear simpler, our results reveal unexpected complexities: *(1)* Human–object occlusion creates ambiguous boundaries, evidenced by the Objects category having the lowest mIoU (79.1% vs. Human’s 86.3%); *(2)* Point density variations manifest uniquely. Sparse hand points versus dense object surfaces, explaining PTv3’s strong baseline performance (80.4% mIoU) as its sequential encoding handles density imbalance effectively.

Second, angular feature fusion provides decisive advantages in multi-positional interactions. Comparing the configuration with angular fusion only (w/o T-Net) to the backbone (Backboned) in Table 2, we observe significant gains: the ±30° category improves from 80.8% to 84.1% (+3.3%), and the ±60° category from 77.7% to 79.7% (+2.0%). These improvements are crucial for robotic arm trajectory planning during oblique approaches. Notably, the Objects category also benefits from angular fusion, increasing from 75.3% to 77.9% (+2.6%), confirming that relative-positional encoding is as critical as object geometry encoding in this application.

Third, the importance of T-Net is highlighted by its removal from the full model: comparing Ours to w/o T-Net, the mIoU of Objects drops by 1.2% (from 79.1% to 77.9%) and Human by 0.9% (from 86.3% to 85.4%), whereas static Others only decline by 0.2% (from 91.7% to 91.5%). This confirms that T-Net is particularly critical for aligning spatial features of moving entities in human–robot handover scenarios.

Finally, the practical deployability of the proposed method remains robust with 78 ms latency (3.2× faster than Swin3D-L), ideal for embedded systems with real-time requirements. Potential limitations include that the ±60° angle situation is still challenging, likely caused by frequent movements during handovers, where human/robot motions create ambiguous geometry signatures. Future work will develop adaptive strategies targeting these high-ambiguity angles.

### 5.2. Comparing Grasp Generation with GraspNet

#### 5.2.1. Experimental Setups

This experiment comprehensively evaluates the grasp performance of our method and GraspNet [26] across multiple human grasp postures and relative angular positions.

**Figure 11:**
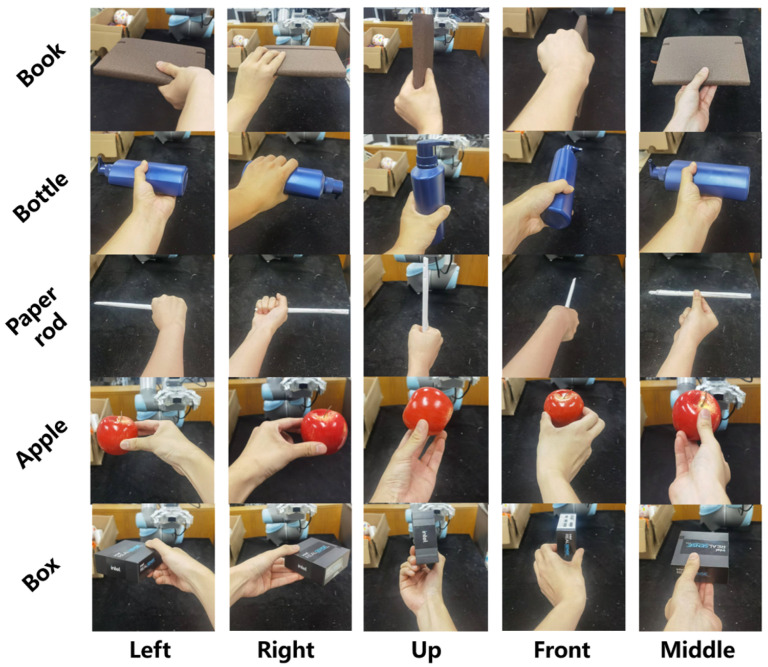
Five representative hand postures are selected in grasping experiments.

- **Baseline method**: GraspNet is a conventional object-based grasp synthesis approach that generates grasp poses based on objects placed on planar surfaces. In contrast, our method optimizes grasp predictions specifically for human-to-robot (H2R) handovers by leveraging real-world data that account for relative human–robot angular positions and motion planning complexity.
- **Algorithmic setup**: Both methods share the same backbone network, a PointNet++ architecture with three encoder and three decoder layers, where the feature dimensions are set to 64, 128, 256 for the encoder and 256, 128, 64 for the decoder. Training is performed on the synthetic dataset described in Section 3.3.1, which comprises 10,800 simulated samples. We use the Adam optimizer with an initial learning rate of 0.0001, a batch size of 15, and train for 50 epochs.
  For our method, the pre-training process follows exactly the same protocol. The key difference lies in the fine-tuning of the optimal grasp selection module: we additionally utilize 2700 real-world samples from the dataset (Section 4.3.2), where only the optimal grasp label is manually annotated, while the grasp poses themselves are predicted by the network (Section 3.3.3 (3)). This design ensures a fair comparison by keeping the underlying grasp generation identical between the two methods.
- **Handover protocol**: A human participant holds each test object using one of the five representative hand postures illustrated in Figure 11 (left, right, up, front, middle). To simulate natural variations in real handovers, the participant is allowed to introduce a slight random rotation of approximately 15° while maintaining the intended posture.
- **Participants and trial design**: The experiment was conducted under normal indoor lighting conditions (Section 4.2) with one healthy participant who signed informed consent. Handovers were performed with the participant standing at five distinct orientations relative to the robot: −60°, −30°, 0°, 30°, and 60°, as illustrated in Figure 3. In total, 250 handover trials were carried out (5 objects × 5 human grasp postures × 2 methods × 5 angles).
- **Test objects**: Five everyday objects are used in the evaluation: a bottle, an apple, a box, a rod, and a book, as shown in Figure 11.
- **Evaluation metric**: The primary metric is the handover success rate, defined as the ratio of successful trials to the total number of valid trials. The precise definition of a successful handover, along with the conditions that constitute failure, follows the protocol established in Section 4.4.2.

#### 5.2.2. Results and Discussion

**Figure 12:**
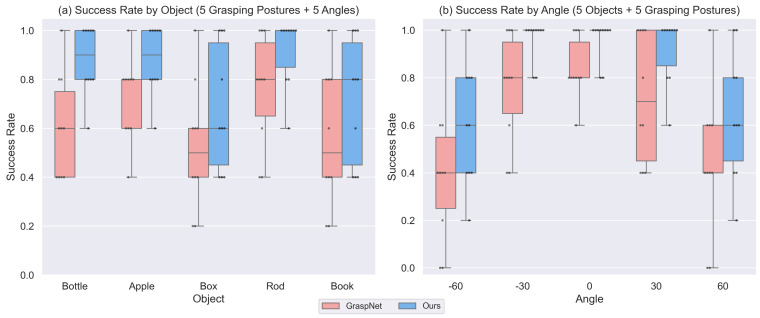
Comparison of grasp success rates between GraspNet and our method across different objects and gripper orientations. (**a**) Boxplots grouped by object type, where each box summarizes the success rates from five grasping postures (left, right, up, front, middle) and five angles. (**b**) Boxplots grouped by relative positional angle, where each box summarizes the success rates from five objects (bottle, apple, box, rod, book) and the five postures. In both panels, the boxes represent the interquartile range (IQR) with the median indicated by a horizontal line, and whiskers extend to the minimum and maximum values.

Quantitative results (Figure 12, Table 4) demonstrate our method’s consistent superiority. The overall success rate for our approach reached 81.6% (102/125) compared with GraspNet’s 63.2% (79/125).

We performed Mann–Whitney U tests to compare the success rates between GraspNet and our method for each object category and each relative angle category (see Section 4.5.1 for details). The tests revealed that our method significantly outperformed GraspNet for the bottle (p=0.026), while differences for apple (p=0.166), box (p=0.258), rod (p=0.130), and book (p=0.248) were not statistically significant. For the angle-wise comparison, a significant improvement was observed only at −30° (p=0.045); at −60° (p=0.165), 0° (p=0.167), 30° (p=0.071), and 60° (p=0.164) the differences did not reach significance. The overall success rate improvement was also significant ($\chi^{2}$ test, p=0.002).

(1) Per-Object Analysis

For object bottle, our method achieved 88% success (22/25) versus GraspNet’s 60% (15/25), with particularly strong improvement in “Front” posture (from 2/5 to 5/5). For object paper rod, our method reached 92% success (23/25) versus 76% (19/25). GraspNet showed particular instability with the box (52% success) due to orientation sensitivity in holding postures (Table 4).

(2) Posture-Wise Analysis

Handovers at relative positional angles ±60° saw 47.6% higher success rates with our approach. At −60°, our method succeeded in 15/25 trials (60%) versus GraspNet’s 10/25 (40%); at 60°, the improvement was more pronounced (16/25 vs. 11/25). Grasping objects held at Front posture improved by 80% under our method (from 10/25 to 18/25). Both methods performed optimally near 0°.

(3) Failure Analysis

GraspNet’s limitations stem from its static-object assumption, leading to inadequate collision consideration in flexible handover orientations and the rigidity being generalized to human grasp variations. While our method demonstrates significant improvements over GraspNet, both approaches share fundamental limitations under extreme handover conditions:

At ±60° relative angles with objects oriented *away* from the robotic workspace (e.g., −60° in Right posture, 60° in Left posture), success rates remain below 20% (GraspNet: 0/10, Ours: 2/10). However, failure analysis of these cases stems from collision avoidance constraints in joint space planning rather than grasp prediction errors.

At the “Up” posture for the “Box” object, both methods struggle at certain angles (GraspNet: 1/5, Ours: 2/5), likely due to misjudgment of shape features caused by perception and instrument accuracy. However, our method still manages to succeed in more instances than the baseline.

### 5.3. Evaluation Perception Pipeline in Adverse Lighting Conditions

#### 5.3.1. Experimental Setup

This experiment evaluates the robustness of our proposed dual-path perception pipeline under challenging illumination conditions. The pipeline integrates two complementary imaging modalities: the fixed ToF-based scene camera and the eye-in-hand stereo camera, detailed in Section 4.1. The primary metric is the handover success rate, defined following the criteria in Section 4.4.2.

(1) Control Groups

To validate the contribution of each modality and the adaptive switching mechanism, we compare three perception strategies:

- **Group A (Adaptive switching—Ours)**: Under normal lighting, the system fuses point clouds from both cameras. When darkness or glare is detected, it autonomously switches to ToF-only perception, deactivating the passive NIR camera.
- **Group B (Scene camera only baseline)**: Relies exclusively on the fixed ToF camera for semantic segmentation and grasp generation under all lighting conditions. This baseline isolates the performance of the active ToF modality.
- **Group C (Eye-in-hand only baseline)**: Uses solely the eye-in-hand passive NIR stereo camera throughout all trials, regardless of lighting. This baseline reflects the degradation of passive stereo under adverse conditions as characterized in Section 4.2.

(2) Lighting Conditions

The three controlled lighting scenarios (normal, dark, glare) follow the specifications defined in Section 4.2, including illuminance levels and the resulting effective point cloud acquisition rates for each camera type. To ensure fair comparison under glare, the directional LED array is always aimed at the primary perception sensor of each group:

- For Group C, the array directly faces the eye-in-hand passive NIR camera, causing overexposure and performance degradation as described in Section 4.2.
- For Groups A and B, the array directly faces the scene ToF camera; the eye-in-hand camera of Group A is fully deactivated during glare trials and provides no data. This imposes identical optical interference on the active imaging link across groups.

**Figure 13:**
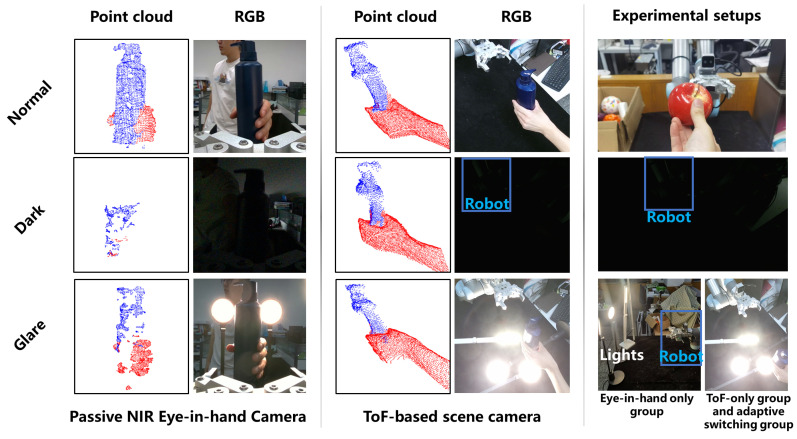
Experimental setups and visual information acquired by cameras in three lighting conditions: dark, glare, and normal.

Figure 13 illustrates the experimental lighting setups and the corresponding point cloud acquisition performance. The left and middle columns show the point cloud data acquired by the two cameras under different lighting conditions, demonstrating their effective acquisition rates. The right column depicts the constructed lighting environments, with the lower-right panel providing a schematic of the glare interference applied to each camera respectively.

(3) Participants and Trial Design

Two healthy adult participants provided written informed consent and performed all handovers. Each participant held the five test objects (bottle, apple, box, rod, book; see Figure 11) using the five representative hand postures (left, right, up, front, middle). To simulate natural variability, a random orientation variation of approximately ±15° was allowed while maintaining the intended posture. The relative angle between participant and robot was fixed at 0°.

For each combination of object, posture, perception group (A, B, C), and lighting condition (normal, dark, glare), each participant completed 1 valid handover. In total, 450 handover trials were carried out (5 objects × 5 postures × 3 groups × 3 lightings × 2 participants).

#### 5.3.2. Results and Discussion

**Figure 14:**
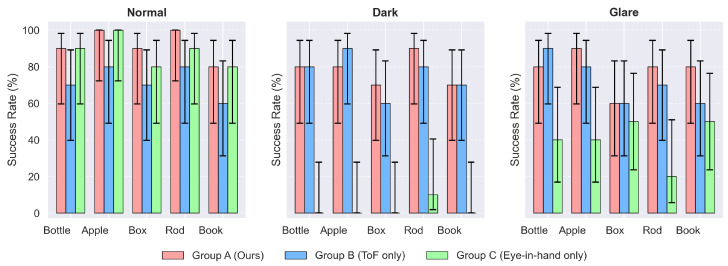
Handover success rates under normal, dark, and glare lighting conditions for five objects and three system groups (Group A: adaptive switching, Group B: ToF only, Group C: eye-in-hand only). Bars represent mean success rates, with error bars indicating 95% Wilson confidence intervals.

Figure 14 visualizes the handover success rates under three lighting conditions for each object and system group, with error bars representing 95% Wilson confidence intervals, providing a detailed view of object-level performance. The overall success rates for each system group are summarized in Table 5. These results unequivocally demonstrate the superior robustness of the proposed method in adverse lighting scenarios. Under normal illumination conditions, all three systems exhibited relatively high performance for most objects, with Group A achieving a 46/50 success rate and Group C yielding a 44/50 success rate.

However, these results changed dramatically in adverse lighting. The performance of Group C collapsed completely under dark conditions (1/50 success rate) and decreased considerably under glare (20/50 success rate). As illustrated in Figure 13, the integrity of point cloud data acquisition from eye-in-hand camera dropped below 10% in darkness due to insufficient light and a degraded signal-to-noise ratio. Moreover, the integrity remained below 40% under glare due to sensor saturation. These results demonstrate that the performance of passive stereo depth cameras varies drastically under different lighting conditions, which highlights the importance of dual-path perception in mutually compensating for each modality’s weaknesses across distinct lighting scenarios.

The proposed system, leveraging camera fusion, maintained high performance across all lighting conditions, achieving a 39/50 success rate in both darkness and glare, outperforming Group B (36/50 in normal, 38/50 in dark, and 36/50 in glare). The slight superiority of Group A over Group B (124/150 vs. 110/150 overall) exists under normal conditions (Group A: 46/50, Group B: 36/50). The key factor for this advantage is that Group A had access to data from the eye-in-hand camera under normal lighting conditions compared with Group B. Therefore, this suggests that the ability to acquire richer features from the eye-in-hand camera under normal illumination enhances grasp performance for irregular objects like boxes and books. Furthermore, participant-introduced random orientation variations (±15°) in the handover trials did not degrade the results, indicating the method’s generalization ability.

However, performance across objects revealed dependencies on object geometry. For instance, Group A excelled with complex objects like the paper rod and apple, but marginally underperformed on the box and bottle in glare. This suggests that while adaptive switching mitigates most lighting challenges, reflective surfaces under glare may still induce minor artifacts in the ToF sensor. Nevertheless, the overall trend remains decisive: adaptive switching achieved an 82.7% total success rate (124/150), surpassing Group B (ToF only, 73.3%, 110/150) and Group C (Eye-in-hand only, 43.3%, 65/150). These results prove that adaptive sensor fusion significantly enhances handover reliability in real-world environments with dynamic illumination, while also highlighting the limitations of unimodal approaches.

Statistical analysis (Section 4.5.2) confirmed these observations: overall success rates differed significantly among groups ($\chi^{2}$ test, p<0.001). Pairwise comparisons revealed that Group A significantly outperformed Group C (p<0.001) and Group B significantly outperformed Group C (p<0.001), while the difference between Group A and Group B was not statistically significant (p=0.070). Under normal lighting, Group A significantly outperformed Group B (p=0.019), and Group A and Group C showed comparable performance (p=0.739); the difference between Group B and Group C approached but did not reach significance (p=0.080). In darkness, Group A and Group B performed similarly (p>0.999), but both significantly exceeded Group C (Fisher’s exact test, p<0.001 for both). Under glare, Group A and Group B again did not differ significantly (p=0.644), yet both significantly outperformed Group C (p<0.001 and p=0.003, respectively). These results quantitatively substantiate the robustness of adaptive dual-path perception and the limitations of unimodal approaches under challenging illumination.

### 5.4. Comparing with Multimodal SOTA Handover Method in Adverse Lighting

To further validate the lighting robustness of our proposed adaptive dual-path handover system, we conducted a controlled comparative experiment with a state-of-the-art (SOTA) multimodal H2R handover framework under the identical hardware and environmental settings as our previous experiments.

#### 5.4.1. Baseline Method

We selected an object-independent H2R handover framework proposed by Rosenberger et al. [5] as the comparison baseline. This work is a representative and widely cited SOTA in the field of H2R handover, which has been extensively validated in subsequent HRI research.

The core pipeline of the baseline method is perceiving the scene via an RGB camera to generate 2D pixel-level masks of the target object and human hand, mapping the masks to aligned depth data to extract the object point cloud, and finally performing handover with an end-to-end grasp generation network.

To ensure absolute fairness of the comparative experiment, we adopted a “modular equivalence strategy” for the baseline framework, which fully retains the core logic of the original paper, with detailed settings as follows:

- **Perception Source**: Consistent with the original paper, only the RGB-D data from the fixed scene camera (Kinect V2, identical to our system’s scene camera) is used as the perception input.
- **Background Filtering**: Based on the depth threshold, we filter out RGB pixels corresponding to depth values outside the range of 0.1–0.75 m, to eliminate background clutter and maximize the performance of subsequent semantic segmentation.
- **2D Semantic Segmentation**: We use the YOLOv8-seg model (a more advanced instance segmentation architecture than the YOLOv3+ResNet used in the original paper) to generate 2D pixel masks of the target object and human hand, which achieves stable segmentation performance under normal lighting.
- **Object Point Cloud Extraction**: The 2D masks generated by YOLOv8-seg are mapped to the aligned depth image of Kinect V2 to extract the object point cloud, which is fully consistent with the core logic of the original paper.
- **Grasp Generation**: The GraspNet model is used for 6-DoF grasp pose prediction, which follows the same principle of the GGCNN-based grasp prediction in the original paper, and uses the exact same training and deployment settings as the GraspNet baseline in Section 5.2 of this paper.

**Figure 15:**
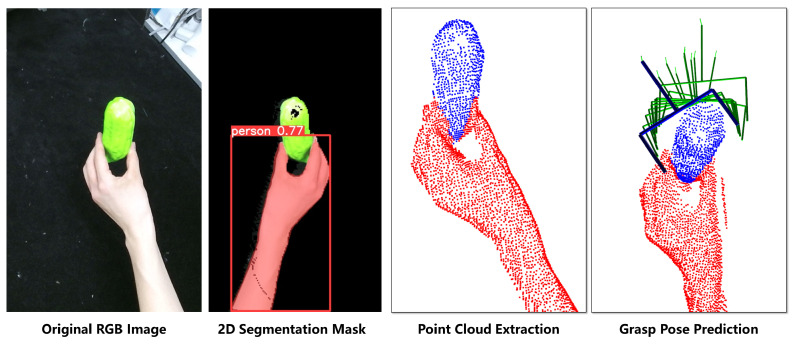
Perception pipeline of the SOTA baseline method, with a demonstration of its stable segmentation and grasp generation performance under normal lighting conditions. From left to right: original RGB image, 2D instance segmentation mask with background filtering, extracted object point cloud, and predicted grasp pose. The blue gripper in the figure represents the best pose predicted by GraspNet.

All other experimental settings, including the hardware platform, lighting conditions, test objects, handover protocol, success criteria, and robot motion control strategy, are exactly the same as our proposed method. This ensures that the only variable between the two groups is the perception and segmentation framework. Figure 15 illustrates the perception pipeline of the baseline SOTA method used for comparison in the experiment.

#### 5.4.2. Experimental Setup

**Figure 16:**
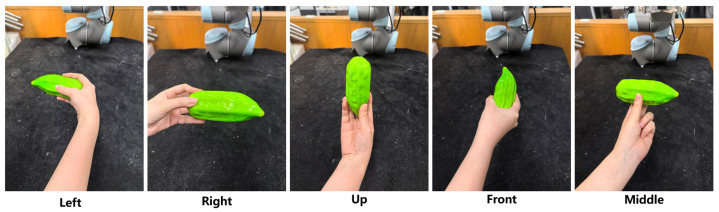
Five representative human hand postures for the test object in the SOTA comparison experiment.

- **Handover Protocol**: Consistent with Section 5.3, participants stand directly opposite the robotic arm (relative positional angle fixed at 0°), hold the object with 5 representative hand postures (left, right, up, front, middle, as shown in Figure 16) and perform handover trials. Participants are allowed to introduce natural ±15° orientation variations of the object to simulate the natural changes in real handover scenarios.
- **Participants and Trial Design**: 2 healthy participants who signed informed consent forms completed all trials. For the two adverse lighting conditions (dark and glare), each participant completed 1 valid handover trial per hand posture for each method. In total, we conducted 40 independent handover trials: 2 participants × 2 lighting conditions × 2 methods × 5 hand postures.
- **Lighting Conditions**: Fully consistent with Section 4.2: the dark condition has a measured illuminance of <3 lux at the camera lens, and the glare condition has a measured illuminance of >930 lux, using the same lighting equipment as Section 5.3.
- **Test Object**: We selected a solid-color plastic fruit as the test object, which has a regular shape and uniform texture. This design provides the most favorable conditions for the pixel-level semantic segmentation of the baseline method, eliminating the interference of object texture and irregular geometry, so that the comparison results can focus on the impact of lighting conditions on the performance of the two methods.
- **Software and Hardware Platform**: Fully consistent with Section 4.1. The YOLOv8-seg model of the baseline method is implemented via the Ultralytics library in Python, with the target category set to “Person” and the confidence threshold set to 0.1, to ensure maximum detection capability in the controlled background.
- **Evaluation and Failure Recording**: The handover success/failure criteria are fully consistent with the definition in Section 4.4.2. For each failed trial, we synchronously record the failure type, which is divided into three categories: (1) Collision between the robot and the human hand/body; (2) Motion planning failure for the predicted target grasp pose; (3) No valid grasp pose output by the system within the valid time window.

#### 5.4.3. Results

The experimental results are summarized in Table 6 and Table 7, which count the number of successful handovers and the failure types of handover trials respectively.

Table 6 shows the handover success times of the two methods under adverse lighting: the SOTA baseline achieved 3 successful trials in glare interference and 0 successful trials in darkness, with a total of 3 successful trials out of 20. Our method achieved 7 successful trials in glare interference and 8 successful trials in darkness, with a total of 15 successful trials out of 20.

The detailed classification of failure phenomena is as follows:

- For our method: all failures are concentrated in collisions between the robot and the human hand/object (5 cases in total), which occurred in both glare (3 cases) and darkness (2 cases).
- For the SOTA baseline: the main failure type is “no valid grasp pose output” (11 cases in total; 1 case in glare and 10 cases in darkness), which leads to zero successful handovers in the dark environment. In addition, 4 failures are caused by collision, and 2 failures are caused by motion planning failure, all of which occurred under glare interference.

**Figure 17:**
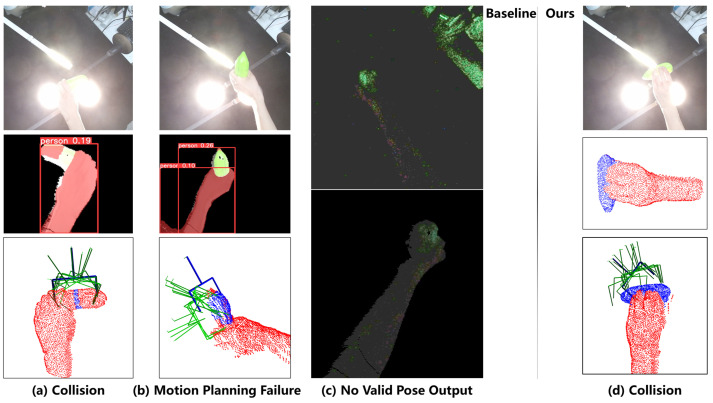
Failure case comparison between the baseline method and our proposed method. Left column: three typical failure types of the baseline method under adverse lighting: (**a**) collision caused by hand segmentation failure, (**b**) motion planning failure caused by incomplete object point cloud, (**c**) no valid grasp output caused by darkness. The right column (**d**) shows a typical failure case of our method, where a collision is caused by grasp prediction error under severe occlusion.

Typical failure cases of the two methods are shown in Figure 17, which provides a visual demonstration of the above failure types.

The handover success rate in this experiment is categorical count data, and all statistical analyses were performed in strict accordance with the pipeline for categorical data specified in Section 4.5.2. Overall, our method significantly outperformed the baseline (15/20 vs. 3/20, $\chi^{2}$ test, p<0.001). Under darkness, the difference was also significant (8/10 vs. 0/10, Fisher’s exact test, p<0.001). Under glare, although our method achieved a higher success rate (7/10 vs. 3/10), the difference did not reach statistical significance ($\chi^{2}$ test, p=0.180).

#### 5.4.4. Discussion

(1) Failure Cause Analysis

11 out of 17 failures of the baseline method are “no valid pose output”, accounting for 64.7% of the total failures. Among them, 10 cases occurred in the handover test in the dark scene (Figure 17c), and 1 case occurred under glare interference. The cause of the failure is that the pixel mask semantic segmentation failed to output valid predictions within the 3-s “Static window”. This reflects the negative impact of glare interference or dark environment on RGB image perception: this impact will not only lead to errors in semantic segmentation details, but also cause the target object to be completely unrecognizable and undetectable. Ultimately, in the task-oriented human–robot handover scenario, the prior information of the object is missing, which leads to the failure of the task. In contrast, our method has no such failures, which indicates that our dual-path perception framework is conducive to implementing human–robot collaboration in scenarios with light interference.

In addition, among the other 6 failures of the baseline method, 2 are caused by “Motion Planning Failure”. This is because the GraspNet model adopted by the baseline method is completely based on its own confidence prediction, without considering the joint constraints or spatial limitations of the robotic arm in H2R handover (Figure 17b). In contrast, our method has no such failures, which indicates that the optimal grasp predicted by the model after learning the spatial features in the training samples is conducive to showing stronger adaptability in space-constrained scenarios.

In the experiment, both methods have failure cases caused by collision (4 cases for the baseline, 5 cases for ours), but the root causes of the failures are completely different:

- Under glare interference, the baseline method is difficult to achieve effective segmentation. The low-quality object point cloud leads to wrong prediction of the grasp prediction model of the baseline, which results in collision failure (Figure 17a).
- For our method, the grasp prediction algorithm is more likely to have deviation when the target object is largely occluded by the human hand (e.g., the “middle” hand posture), which leads to the deviation of the grasp pose and collision failure (Figure 17d). Notably, this failure is evenly distributed in glare and dark environments, and has no significant correlation with lighting conditions, which further verifies the lighting robustness of our dual-path perception framework.

(2) Scene Adaptability Analysis

Based on the above data and failure analysis, this controlled comparative experiment fully verifies that our proposed dual-path perception system solves the limitation of RGB-based multimodal SOTA H2R handover methods in object recognition and perception under adverse lighting conditions. In addition, the grasp selection module optimized for H2R handover scenarios is more adaptable to the spatial constraints and dynamic scenes of human–robot handover than the general grasp model, and effectively avoids the problem of motion planning failure.

The conclusion of this experiment is completely consistent with the results of the previous semantic segmentation, grasp generation, and full-system lighting robustness experiments, and multiple sets of experiments cross-verify the stability and generalization of the proposed method.

However, as shown in the experimental results, the proposed method still has a probability of collision failure caused by grasp prediction deviation in the scenario where the object is largely and severely occluded by the human hand. Therefore, in future work, we will introduce the geometric prior of the hand–object occlusion relationship into the segmentation model to optimize the object boundary segmentation accuracy in severely occluded scenarios, thereby improving the robustness of grasp pose prediction.

## 6. Conclusions

As a core task of human–robot collaboration, human-to-robot (H2R) handover faces severe challenges from complex lighting environments, which often cause fatal performance degradation of pure RGB-based perception systems. To address this issue, this paper proposes a robust H2R handover system with a dual-path perception architecture as the core, combined with task-optimized semantic segmentation and grasp generation algorithms, achieving stable and precise handover operations under overexposed, low-light, and other adverse lighting conditions.

The core innovations of this work are targeted at the specialized pain points of H2R handover scenarios. The dual-path perception architecture realizes parallel lighting-robust feature extraction and semantic segmentation, which effectively solves the problem of segmentation accuracy drop caused by lighting interference and hand–object occlusion; the task-oriented optimized grasp algorithm incorporates an optimal selection strategy, significantly improving generalization ability for multi-angle handovers under the geometric constraints of the robotic arm. Systematic experiments verify that the proposed system maintains a handover success rate of 82.7% in complex lighting environments, with a mIoU score in segmentation improved by 3.26 percentage points compared with mainstream baseline methods, fully demonstrating the effectiveness and robustness of the framework.

The current system still has limitations. It focuses on the pre-grasp perception and planning pipeline, which limits its adaptability to dynamic disturbances from human limbs during the handover process. Related work has explored impedance learning for this scenario [31]. Based on this idea, the robust perception strategy proposed in this paper can be effectively transferred to compliant manipulation in complex lighting scenarios. Meanwhile, its adaptive impedance learning method for unknown contact environments can supplement the fault handling capability of the system. It can effectively cope with dynamic contact disturbances and potential collision risks during handover, and improve the fault tolerance and safety of H2R interaction. Future work will focus on introducing dynamic trajectory prediction to handle fast-moving objects, further integrating the proposed framework with impedance learning-based compliant control methods, and exploring multimodal perception fusion with force/tactile sensors to achieve more natural and safer H2R handover.

## Figures and Tables

**Table 1:** Performances of Semantic Segmentation Models on Our Dataset.

Methods	mIoU (%)	mAcc (%)	Test Loss	Inference Latency (ms)
Ours	84.4±1.7	90.3±1.5	0.167±0.011	78±2
Pointnet++ [28]	79.9±3.0	85.3±2.8	0.261±0.053	247±5
Swin3D-L [29]	81.1±2.8	86.5±2.6	0.223±0.042	413±8
SphereFormer [30]	75.9±3.6	82.7±3.3	0.303±0.044	460±10
PTv3 [20]	80.4±1.6	87.6±1.5	0.199±0.011	56±2

**Table 2:** Per-category and overall mIoU (%) in the ablation study.

Category	Backboned	w/o Angular	w/o T-Net	Ours
Others	89.8±1.4	90.2±1.5	91.5±1.3	91.7±1.2
Human	81.7±2.0	83.1±2.2	85.4±1.9	86.3±1.8
Objects	75.3±2.3	76.8±2.5	77.9±2.1	79.1±2.0
Relative angle (0°)	85.1±1.7	86.6±1.9	87.0±1.6	87.7±1.5
Relative angle (±30°)	80.8±2.1	82.3±2.3	84.1±2.0	85.2±1.9
Relative angle (±60°)	77.7±2.6	78.2±2.8	79.7±2.4	81.9±2.2
Mean mIoU	80.4±1.6	81.5±1.9	82.9±1.5	84.4±1.7

**Table 3:** Angle classification accuracy (%).

Angle Category	Accuracy
0°	94.8±2.5
±30°	86.3±4.0
±60°	89.7±3.3
Overall Accuracy	90.3±3.1

**Table 4:** Summary of grasp success counts (successful trials/total trials) for GraspNet and our method, grouped by object, relative positional angle, and human gripping posture.

Category	GraspNet		Ours	
	**Success**	**Rate (%)**	**Success**	**Rate (%)**
**By object**				
Bottle	15/25	60.0	22/25	88.0
Apple	18/25	72.0	22/25	88.0
Box	13/25	52.0	17/25	68.0
Rod	19/25	76.0	23/25	92.0
Book	14/25	56.0	18/25	72.0
**By angle**				
−60°	10/25	40.0	15/25	60.0
−30°	19/25	76.0	24/25	96.0
0°	21/25	84.0	24/25	96.0
30°	18/25	72.0	23/25	92.0
60°	11/25	44.0	16/25	64.0
**By posture**				
Left	17/25	68.0	20/25	80.0
Right	17/25	68.0	21/25	84.0
Up	16/25	64.0	20/25	80.0
Front	10/25	40.0	18/25	72.0
Middle	19/25	76.0	23/25	92.0
**Overall**	79/125	63.2	102/125	81.6

**Table 5:** Overall Handover Success Performance under Different Lighting Conditions.

System Group	Normal	Dark	Glare	Overall
Group A (Ours)	46/50	39/50	39/50	124/150
Group B (ToF only)	36/50	38/50	36/50	110/150
Group C (Eye-in-hand)	44/50	1/50	20/50	65/150

**Table 6:** Handover Success Performance under Adverse Lighting Conditions.

Method	Glare Interference	Darkness	Overall Success Rate
SOTA Baseline [5]	3/10	0/10	3/20 (15.0%)
Ours	7/10	8/10	15/20 (75.0%)

**Table 7:** Failure Type Statistics under Adverse Lighting Conditions.

Method	Collision	Motion Planning Failure	No Valid Pose Output	Total Failures
SOTA Baseline [5]	4	2	11	17/20
Ours	5	0	0	5/20

## Data Availability

The original contributions presented in this study are included in the article; further inquiries can be directed to the corresponding author.
